# Evaluation of an interferon-gamma release assay for early detection of lumpy skin disease virus infection and vaccination in cattle

**DOI:** 10.1128/spectrum.02939-24

**Published:** 2025-03-10

**Authors:** Nina Kresic, Wannes Philips, Andy Haegeman, Nick de Regge

**Affiliations:** 1 Sciensano, Unit Exotic and Vector Borne Diseases (ExoVec) 54513 , Brussels, Belgium; National Chung Hsing University, Taichung, Taiwan, China

**Keywords:** immunity, capripoxviruses, immunoassays

## Abstract

Lumpy skin disease virus (LSDV) causes a nodular dermatitis in cattle and has high economic consequences in affected areas. Detection of LSDV exposure mostly relies on the humoral immune response, while the cell-mediated immune (CMI) response, an important hallmark of the immune reaction to LSDV, is neglected. We collected samples during 3 weeks post-vaccination of cattle with a Neethling-based live attenuated vaccine (LAV) and during 4 weeks post-LSDV infection under experimental conditions to i) investigate the development of the CMI response, ii) optimize an interferon-gamma release assay (IGRA) by comparing two matrices (whole blood and PBMCs) and different stimuli, and iii) evaluate the usefulness of an IGRA for detection of infection and vaccination. The CMI response to the Neethling LAV was detectable in all animals from 10 days post-vaccination, and importantly, preceded the detection of antibodies. A uniform CMI response to infection was already detected at its plateau level at 7 days post-infection in all animals and also preceded antibody detection. Whole blood and PBMCs allowed efficient IFN-γ secretion, but the IFN-γ response was significantly higher in PBMCs. Heat-inactivated antigen proved to be the stimulus of choice for LSD IGRA, providing increased sensitivity of the test and allowing its performance under BSL2 conditions. Under several conditions, LSDV IGRA showed a specificity of up to 100%. The LSD IGRA could be a powerful immunoassay for early detection of LSD and post-vaccination monitoring, making it worthwhile to explore its diagnostic characteristics in more animals and under field conditions.

**IMPORTANCE:**

The immune reaction to lumpy skin disease virus (LSDV) infection or vaccination is currently assessed with serological tests prone to suboptimal sensitivity, long processing time, and the necessity of biosafety level (BSL) 3 laboratories. Furthermore, the delayed or absent seroconversion indicates a need for an alternative immunoassay detecting immune reactions to LSDV exposure applicable in BSL2 settings. Seeing the known importance of cell-mediated immune (CMI) response against poxvirus infections, we evaluated the suitability of the interferon-gamma release assay (IGRA) test for detection of LSDV infection and vaccination. IGRA allowed early detection of the CMI response to LSDV infection (within 7 days) and vaccination (within 10 days) with a Neethling-based live attenuated vaccine, and the CMI response preceded the detection of seroconversion. Whole blood and heat-inactivated antigen increased IGRA sensitivity, making it suitable for application in BSL2 laboratories. This assay overcomes the downsides of currently available immunoassays, and these results encourage its further evaluation.

## INTRODUCTION

Lumpy skin disease (LSD) is caused by the lumpy skin disease virus (LSDV), which belongs to the *Capripoxvirus* (CaPV) genus belonging to the family *Poxviridae*. It is a nodular dermatitis affecting cattle and water buffalos and is classified as a notifiable disease by the World Organisation of Animal Health (WOAH) (1, 2). Indications of LSDV infections of other susceptible hosts have been found in seroprevalence studies, experimental infections, and case reports (2–4).

For a long time, LSD was regarded as endemic in the African continent. Over time, it has reached the Middle East, causing outbreaks in Israel in 2012 and Turkey in 2013, and subsequently, the disease spread to the Balkans in 2015. In 2017, recombinant strains were identified and spread across Asia where currently, the classical clade 1.2 strains and recombinant clade 2 strains are circulating (2, 3, 5–7) with high economic consequences.

Regardless of the causative strain, clinical LSD is presented as a mild-to-serious illness with a mortality of up to 5% in cattle (1). The onset of the nonspecific infectious syndrome (temperature, lacrimation, diarrhea, nasal discharge, reduced appetite, and decrease in milk production) is followed by nodular dermatitis with the firm nodules, progressing over time to necrosis, a main characteristic of LSD. Nodules appear all over the animal or at predilection places with thin and delicate skin (eyelids, perineum, udder, scrotum, and ears). Subclinical forms of the disease are common, whereby animals appear healthy and without nodules, but productive virus replication can be detected with laboratory analyses, and subclinical animals contribute to disease transmission (8).

The disease can be controlled and eradicated via the interplay of clinical surveillance, vaccination with live attenuated vaccines, and early detection of LSDV antigen and antibodies (9–15). Antigen detection relies on numerous diagnostic methods with high specificity and sensitivity. However, challenges to their performances in the field can be posed by disease and virus biology, such as subclinical LSD, intermittent virus occurrence in blood and nasal secretions, and the occurrence of recombinant strains circulating in the field, requiring prompt updating of the available DIVA methodologies.

Disease-specific antibodies produced by the humoral immune response can be detected with enzyme-linked immunosorbent assays (ELISA) and immunoperoxidase monolayer assays (IPMA), while neutralizing antibodies are detected with virus neutralization tests (VNT) (1). Unfortunately, the moment of detection of seroconversion is test-dependent. IPMA, although time-consuming, proved to be very sensitive and detects antibodies at 8–12 days post-vaccination (dpv) or days post-infection (dpi) (11). ELISA tests detect seroconversion relatively late, from 17 to 21 dpv or dpi, and only some vaccinated animals become ELISA-positive. Late or even absent detection of seroconversion in ELISA decreases its diagnostic sensitivity and consequently, utility.

Given the above-mentioned constraints of molecular and serological testing tools, there is still a need for a fast, easy, and accurate diagnostic test that allows early detection of the immune reaction to LSDV antigens. In this respect, we here explored the usefulness of detecting an LSDV-specific cellular immune response by measuring interferon-gamma (IFNg) release by primed immune cells upon restimulation.

Experimental infections (11–13) and field studies have demonstrated that the LSDV immune response is predominantly cell-mediated. The cell-mediated immune (CMI) response is initiated with immune cell priming post-vaccination or post-infection. Administered vaccine antigens or viruses that break the first line of defense are taken up by professional antigen-presenting cells (APCs). These cells present antigen peptides on their major histocompatibility complexes (MHC) I and MHC II to naïve lymphocytes within secondary lymphoid organs, draining the site of vaccination or infection (14). Once T cells have received the necessary stimulation by APCs, they can proliferate to mount a strong immune response. Proliferating primed T cells develop different effector functions, and a small part differentiates into long-lived memory cells. Both are circulatory and can be detected in blood. Currently, little is known about the evolution of LSDV-specific immune cells.

The CMI response can be measured with immunoassays measuring IFN-γ secreted by immune cells such as IGRA or with tests measuring the number of antigen-specific IFN-γ-producing cells (14), such as ELISpot.

IGRA is an immunoassay designed to detect specific IFN-γ release upon re-exposure of sensitized immune cells to live or inactivated antigens or synthetic peptides *in vitro*. In the second step of the test, the amount of IFN-γ released is measured using commercially available IFN-γ ELISAs. IGRA was first used in the field of tuberculosis (TB) diagnostics (15, 16), but today has found its place for the detection of previous exposure or active infection to other diseases as well (17–19). TB IGRA tests are already commercially available, with a new version being approved (20).

IGRA assays have already been used to detect LSDV-specific IFN-γ release in the context of experimental LSDV infection and upon vaccination. It has been shown that the IFN-γ response in LSDV-sensitized animals can be detected as early as 5 dpi (12). Haegeman *et al*. demonstrated that a medium-to-strong IFN-γ response to LAV and inactivated LSDV vaccines could still be detected at 12 and 18 months post-vaccination (11). These results indicate that an LSD IGRA could be a promising tool for the early detection of LSD-infected or vaccinated animals. Accordingly, we further explored in detail the development of the CMI response after LSDV infection and vaccination, optimized the setup of the IGRA test, and evaluated the usefulness thereof for early detection of infection and monitoring of vaccine-induced immunity.

## MATERIALS AND METHODS

### Experimental setup and ethical approval

Samples used in this study were collected during the vaccine effectiveness study of Phivax Neethling-based live attenuated vaccine (LAV) for LSD (21). The study was performed in 6-month-old bulls (*Bos taurus*) without any signs of disease and seronegative for LSDV, bluetongue virus (BTV), and bovine viral diarrhea virus (BVDV). A total of twelve (12) animals participated in the experiment, which included seven animals in the vaccinated group (VAC; *n* = 7) and five in the infected group (INF, *n* = 5). After an acclimatization period of 7 days, vaccinated animals were followed for 3 weeks post-vaccination and infected animals for 4 weeks post-infection.

Animals were vaccinated with a Neethling-based LAV (Phivax LSD, Phibro) administered subcutaneously (s/c) following the manufacturer’s instructions. The animals from the INF group were inoculated with a clade 2.5 recombinant LSDV strain isolated from an outbreak in Vietnam at a titer of 10^6.1^ TCID50/mL by intravenous (i/v) administration into the vena jugularis (3 mL) and intradermal administration (i/d) (1 mL) into two spots on each side of the neck (250 µL per application), giving the total inoculation dose of 5 × 10^6^ TCID_50_. Samples were collected on 0, 3, 7, 10, 14, 17, and 21 dpv/dpi and 24 and 28 dpi.

The animal experiment was performed in the BSL3 facilities of Sciensano according to the European Union and Belgian regulations on animal welfare in experimentation. The protocol was approved by the joined Ethical Committee of Sciensano, authorization number 20230627–01.

### Blood sampling

Fresh whole blood (WB) samples were collected twice a week from the jugular vein into heparin blood tubes using a vacuum system and transported at room temperature to the laboratory. Heparinized WB samples were used for WB IGRA and PBMC isolation (described below) within 4 hours post-sampling.

Additionally, blood sample for serology was collected at the time points to evaluate seroconversion using the enzyme-linked immunosorbent assay (ELISA), virus neutralization test (VNT), and immunoperoxidase monolayer assay (IPMA).

### PBMCs recovery from whole-blood samples

For each animal and time point, PBMCs were isolated from 18 mL of undiluted blood, layered onto 15 mL of sterile Histopaque-1077 (Sigma-Aldrich, United States). After 30 minutes of centrifugation at 920 rcf with the lowest acceleration and brake centrifuge settings (1), the buffy coat was formed as a well-defined layer in between blood plasma on the top and Histopaque-1077 below. A whitish cloud made of PBMCs was carefully aspirated with a 2 mL Pasteur pipet without disturbing the layers and placed into a sterile 50 mL Falcon tube (Fisher Scientific, United States). The remaining autologous plasma, as a source of important costimulatory molecules, was collected into a sterile 15 mL Falcon tube (Fisher Scientific, United States) as a medium for PBMC cultivation in IGRA. Recovered PBMCs were subjected to two rounds of washing in sterile PBS (Gibco, United States) at 600 rcf for 5 minutes. Finally, pelleted PBMCs were gently brought back to their natural medium by resuspending them in 5 mL of homologous plasma. All manipulations were performed at room temperature.

### PBMC quality control

To check the success of our PBMC isolation and their viability, a 50 µL aliquot of PBMCs in autologous plasma was taken, stained with trypan blue (ratio 1:1), and counted in the Thoma chamber. Cells were counted in one medium square with 16 small squares. The cell concentration per milliliter of suspension was determined according to the following formula:

𝐶𝑒𝑙𝑙 𝑐𝑜𝑛𝑐𝑒𝑛𝑡𝑟𝑎𝑡𝑖𝑜𝑛 𝑝𝑒𝑟 𝑚𝑙 = 𝑛𝑢𝑚𝑏𝑒𝑟 𝑜𝑓 𝑃𝐵𝑀𝐶𝑠 𝑝𝑒𝑟 𝑚𝑒𝑑𝑖𝑢𝑚 𝑠𝑞𝑢𝑎𝑟𝑒 𝑥 𝑓𝑎𝑐𝑡𝑜𝑟 𝑜𝑓 𝑑𝑖𝑙𝑢𝑡𝑖𝑜𝑛 𝑥 0.25 𝑥10^6^

### LSD IGRA stimuli

During the LSDV IGRA described in the paragraph below, each sample (both WB and PBMCs) was exposed to the following stimuli, which were all added at 100 µL/well.

### Baseline control

Baseline levels of IFN-γ production were monitored in wells restimulated with only PBS 1 x (Gibco, United States).

### Positive control

The functionality of IFN-γ-producing cells in different matrices was monitored in each assay by restimulation with the polyclonal mitogen, pokeweed (PWM) (160 ng/mL).

### Live virus

The classical clade 1.2 field strain LSD/OA3-Ts.MORAN, kindly provided by the Kimron Veterinary Institute, Israel, and the Israeli Veterinary Services at titer 10^5.8^ TCID50/mL, and the recombinant 2.5 LSDV strain LSD/OA3-Ts.ZOL isolated from a skin sample originating from an outbreak in Vietnam, at titer 10^6.2^ TCID50/mL served as live antigens for restimulation in IGRA.

Both strains were grown and titrated on OA3-Ts cells (ATCC-CRL-6546) aliquoted and stored to be used as live stimulus and for the production of UV-irradiated and heat-inactivated IGRA antigens.

In brief, OA3-Ts were grown in a standard cultivation medium made of high-glucose DMEM (Fisher Scientific, Merelbeke, Belgium) supplemented with 10% gamma-irradiated fetal bovine serum (FBS) certified free from live BVDV (Thermo Fisher Scientific; Belgium), amphotericin B (250 mg/mL, Thermo Fisher Scientific; Merelbeke, Belgium), and penicillin/streptomycin (Thermo Fisher Scientific; Belgium).

Typically 24–48 hours post-seeding, at a confluency of at least 90%, the cell monolayer and flask walls were carefully washed 3 x with 30 mL of sterile PBS (Gibco) to remove all traces of the OA3-Ts medium and prepare the monolayer for infection.

Infection was done with LSDV stocks prepared in virus medium composed of high-glucose DMEM (Fisher Scientific, Merelbeke, Belgium), amphotericin B (250 mg/mL, Thermo Fisher Scientific; Merelbeke, Belgium), and penicillin/streptomycin (Thermo Fisher Scientific; Belgium) without FBS.

Infection was done at a multiplicity of infection 0.1 and incubated for 2 hours at 37°C in the presence of 5% CO_2_ and 90% humidity. After incubation, the inoculum was washed 3 x with 30 mL of sterile PBS (Gibco, Thermo Fisher Scientific), and 50 mL of the virus medium without FBS was added. This approach aimed to prepare pure LSDV stocks in a medium free from FBS as a source of different contaminants.

Infected monolayers were inspected daily for the infection progress, and when 75%–80% of cells were affected, typically 72–96 hours post-infection, cell culture flasks were frozen at −80°C. To release the intracellular virus, frozen bottles were subjected to a fast thawing procedure with shaking, and the flask content was transferred into 50 mL sterile Falcon tubes and centrifuged at 1900 x *g* for 10 minutes. The collected clarified supernatant was well homogenized, aliquoted, and stored to be used as a challenge virus and for the production of heat- and UVC-inactivated IGRA antigens. Quality control testing was performed regularly on produced virus stocks. This included testing for the presence of different viruses: foot and mouth disease virus, BTV, and BVDV and checking for bacterial contamination.

### UVC-irradiated virus

To produce UVC-inactivated antigen, recombinant clade 2.5 LSDV strain, grown as described above, was exposed to UVC (254 nm) light irradiation by a UVpro WDS 20 UVC lamp (Nivonic, Germany). Aliquots of 1 mL were distributed into 24-microwell plates and placed under the UVC lamp at an approximately 10 cm distance for 5 minutes in a wooden box. The calculated dose of irradiation is 210 j/m^2^. The UVC-irradiated virus was collected, well homogenized, and stored in labeled sterile cryotubes at −80°C until used in IGRA.

### Heat-inactivated virus

Recombinant clade 2.5 LDSV strain, grown as described above, was heat-inactivated at 56°C for 4.5 hours with occasional vortexing, well homogenized, and stored in labeled sterile cryotubes at −80°C until used in IGRA.

### Interferon-gamma release assay (IGRA)

An IGRA measures IFN-γ release by sensitized circulatory immune cells upon *in vitro* restimulation with live or inactivated LSDV antigens. The test is performed in two steps. First, immune cells present in WB or PBMCs are mixed with the antigen and incubated overnight at 37°C + 5% CO_2_ for 20 hours (+/–2 hours). In the second step, collected supernatants are tested with commercially available ELISAs to determine the amount of released IFN-γ. A detailed description of the test setup is described below.

We compared LSD IGRA using either WB or freshly isolated PBMCs. WB proved to be a convenient IGRA matrix widely used in the diagnostics of bovine tuberculosis (22). Purified PBMCs cultivated in autologous plasma were chosen as key players in mounting IFN-γ.

### Whole-blood LSD IGRA

A previously described WB IGRA (23) was slightly modified regarding the virus strains used for WB restimulation. Freshly collected heparinized WB in the volume of 1.5 mL was placed in six wells of a 24-well plate (Nunc, Thermo Fisher Scientific, United States), giving a total volume of 9 mL, as needed per animal.

After the addition of 100 µL stimulating antigens, in corresponding wells, plates were shaken for 5 minutes on a plate shaker at room temperature to allow proper distribution of antigens as a prerequisite for a successful reaction. Plates were placed in an incubator at 37°C + 5% CO_2_ and 90% humidity for 20 hours (+/–2 hours). The next day, plates were centrifuged at 500 g for 10 minutes, and the supernatant, typically around 700 µL, was collected into Micronics tubes to be used in IFN-γ ELISA (ID Vet, Montpellier, France) according to the manufacturer’s instructions (protocol 2).

### Fresh PBMC LSD IGRA

The volume of the 417 µL PBMC suspension in autologous plasma, equivalent to the number of PBMCs present in 1,5 mL of whole blood, was distributed in six wells of a 24-microwell plate and stimulated with 100 µL of the stimuli. We also added 183 µL of the seeding medium to achieve a total of 700 µL to resemble the volume of WB plasma typically obtained in WB IGRA. The plates were shaken on a plate shaker for 1–2 minutes to allow antigen distribution among the cells and incubated at 37°C + 5% CO_2_ and 90% of humidity for 20 (+/–2) hours. The next day, plates were centrifuged at 500 g for 10 minutes, and the supernatant was collected into Micronics tubes to be used in IFN-γ ELISA (ID Vet, Montpellier, France) according to the manufacturer’s instructions (protocol 2).

### ELISA

The IFN-γ ELISA kit from IDVET (ID Screen Ruminant IFN-g) was used to detect the presence of IFN-γ in the supernatants of the IGRA test, following the manufacturer’s instructions. This sandwich ELISA detects bovine, ovine, and caprine IFN-γ. Uniprot reports 90% sequence identity for IFNg for different members of family Bovidae. The wells of the ELISA plate are coated with an anti-ruminant monoclonal IFN-γ antibody. *In vitro-*secreted IFN-γ obtained in IGRA specifically binds to the coated monoclonal IFNg antibody and is detected with a conjugate containing anti-ruminant IFN-γ monoclonal antibody labeled with horseradish peroxidase. The binding is visualized upon addition of the TMB substrate.

The Double Antigen ELISA kit from IDVET (ID Screen Capripox Double Antigen Multi-species) was used to detect the presence of LSDV-specific antibodies in serum samples, following the instructions described by the manufacturer.

### Virus neutralization test

The virus neutralization test (VNT) was carried out as described previously (24). In brief, serum samples collected on 7, 10, 17, and 21 dpv/dpi and 28 dpi were titrated against a constant titer of capripoxvirus of 100 TCID_50,_ and the VNT procedure was adapted for visualization by IPMA (24).

### Immunoperoxidase monolayer assay

Immunoperoxidase monolayer assay (IPMA) was performed as described previously (24). In brief, IPMA plates were prepared using LSDV strain Neethling. Serum samples collected in the post-vaccination and post-infection periods were analyzed along with positive and negative reference serum controls.

### Specificity

The specificity of the assay was determined by analyzing samples collected on 0 dpv stimulated with either live, inactivated, and irradiated LSDV.

### Sensitivity

The evaluation of sensitivity of the test was done on reference samples obtained in the vaccine safety and efficacy trial (21). Animals from different groups were classified as follows:

- true positives (TP): animals in direct contact with antigens either by vaccination or infection,
- true negatives (TN): animals that have not been in contact with LSDV before

Thus, all animals in the vaccinated and infected groups once they received vaccination or after they were challenged were TP, while naïve animals (animals before vaccination on 0 dpv) were TN. TN with detectable IFN-γ levels were designated as false positives (FP), while TP without detectable IFNg levels were designated as false negatives (FN). The sensitivity and specificity of the test were determined according to the formulas: TP/(TP +FN) ×100, and TN/(FP +TN) ×100, respectively.

### Statistics

Post-vaccination and post-infection IFN-γ values from stimulated WB and PBMCs were checked for normality with the Kolmogorov–Smirnov test.

#### Matrix evaluation

Average post-vaccination and post-infection plateau IFN-γ levels in response to each of the four stimuli were compared between WB and PBMCs with a nonparametric Mann–Whitney test.

#### Uniformity of the IFN-γ response

The uniformity of response obtained from PBMCs and WB to the four stimuli was analyzed by determining the coefficient of variation (CV). The CV (%) at each time point was determined as the ratio between the standard deviation (SD) and the mean of seven (VAC) biological replicates at 7, 10, 14, 17, and 21 dpv and five (INF) biological replicates at 7, 14, 17, 21, and 28 dpi.

The mean CVs obtained during the plateau phase (10–17 dpv and 10–28 dpi) in response to the four stimuli in each matrix separately were compared with a nonparametric Kruskal–Wallis test.

The mean CV obtained during the plateau phase in response to all the four stimuli were compared between WB and PBMCs in the post-vaccination period (multiple Mann–Whitney test) and post-infection period (multiple Mann–Whitney test).

#### Stimulus evaluation

We analyzed performances of different stimuli post-vaccination (7, 10, 14, 17, and 21 dpv) and post-infection (7, 10, 14, 17, 21, and 28 dpi). Differences in IFN-γ secretion levels between the four stimuli were evaluated for each matrix separately. We also evaluated the differences between live and inactivated and irradiated stimuli within each matrix. These analyses were done with *t*-test and Mann–Whitney tests.

A difference was considered significant when *P* < 0.05. Graphical presentation and statistical analysis were performed with GraphPad Prism 9.

## RESULTS

### PBMC quality control and yield

**Fig 1:**
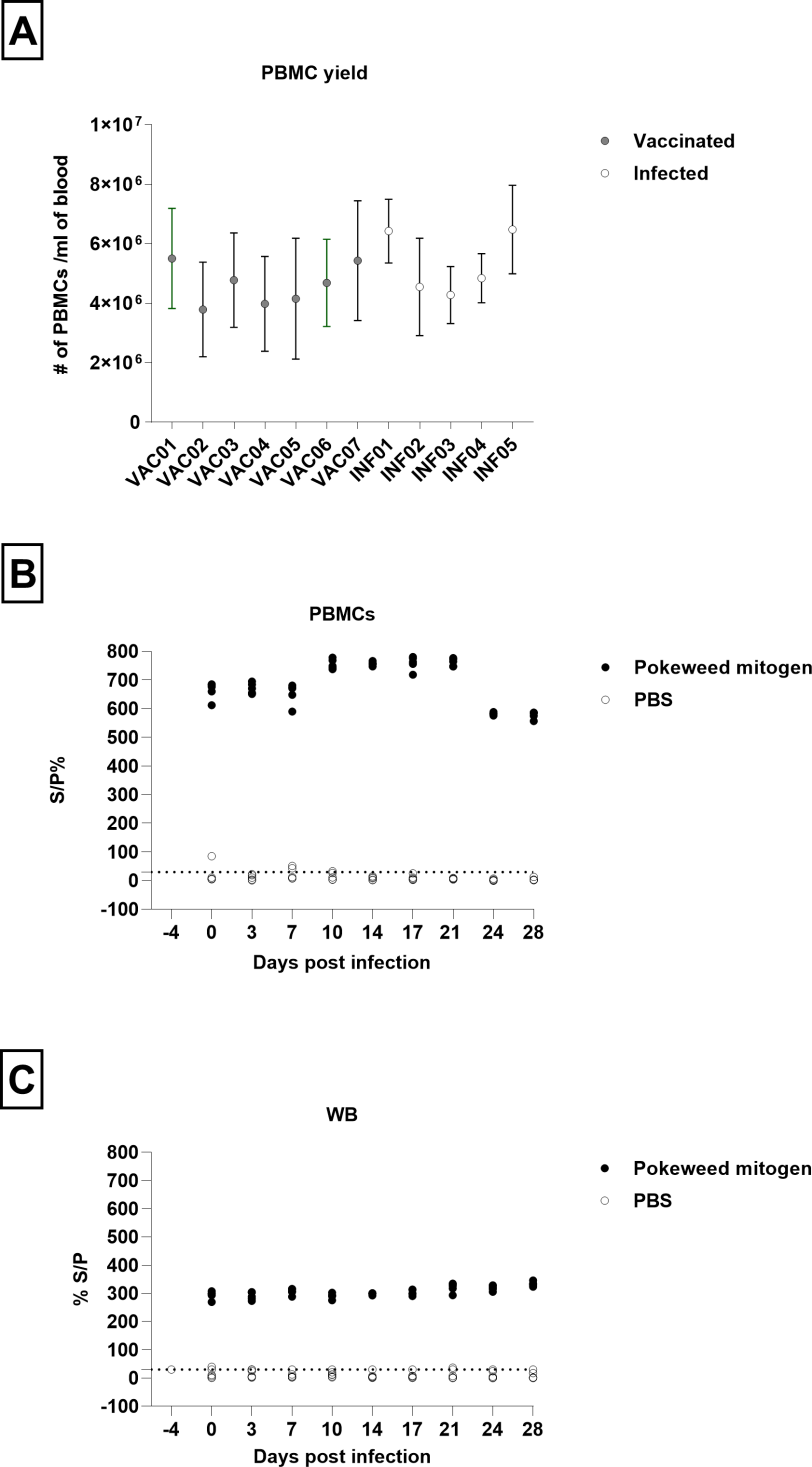
Average number of PBMCs isolated from animals in post-vaccination and post-infection period (A). Interferon gamma (IFNg) release in response to polyclonal mitogen (PWM) as a measure of PBMCs functionality (B and C). Baseline IFNg levels were monitored in PBMCs and WB after restimulation with phosphate buffer saline (PBS) (B and C). Whole blood and PBMCs samples were collected from animals in post-infection period (B and C).

The post-recovery viability of PBMCs was completely preserved, and the average PBMC yield in all animal groups throughout the trial period was within the reference range (2.5–7.5 × 10^6^/mL) (25) for bovine white blood cells (Fig. 1A). The high viability of these fragile primary cells post-recovery supports the suitability of the applied isolation procedure. Indeed, high levels of IFN-γ released upon polyclonal mitogen stimulation and lack of IFN-γ response upon restimulation with PBS (Fig. 1B and C) are good proof of the preserved functionality of the immune fraction within WB and of PBMCs. We additionally explored the baseline levels of IFN-γ during infection using samples obtained after restimulation with PBS. Throughout the trial, LSD IGRA was positive in 3 out of 49 (6.1%) samples in WB and 4 out of 49 (8.1%) samples in PBMCs, indicating the absence of continuous baseline presence of IFN-γ levels during LSDV infection.

### Vaccine-elicited cell-mediated immune response

#### General observations

**Fig 2:**
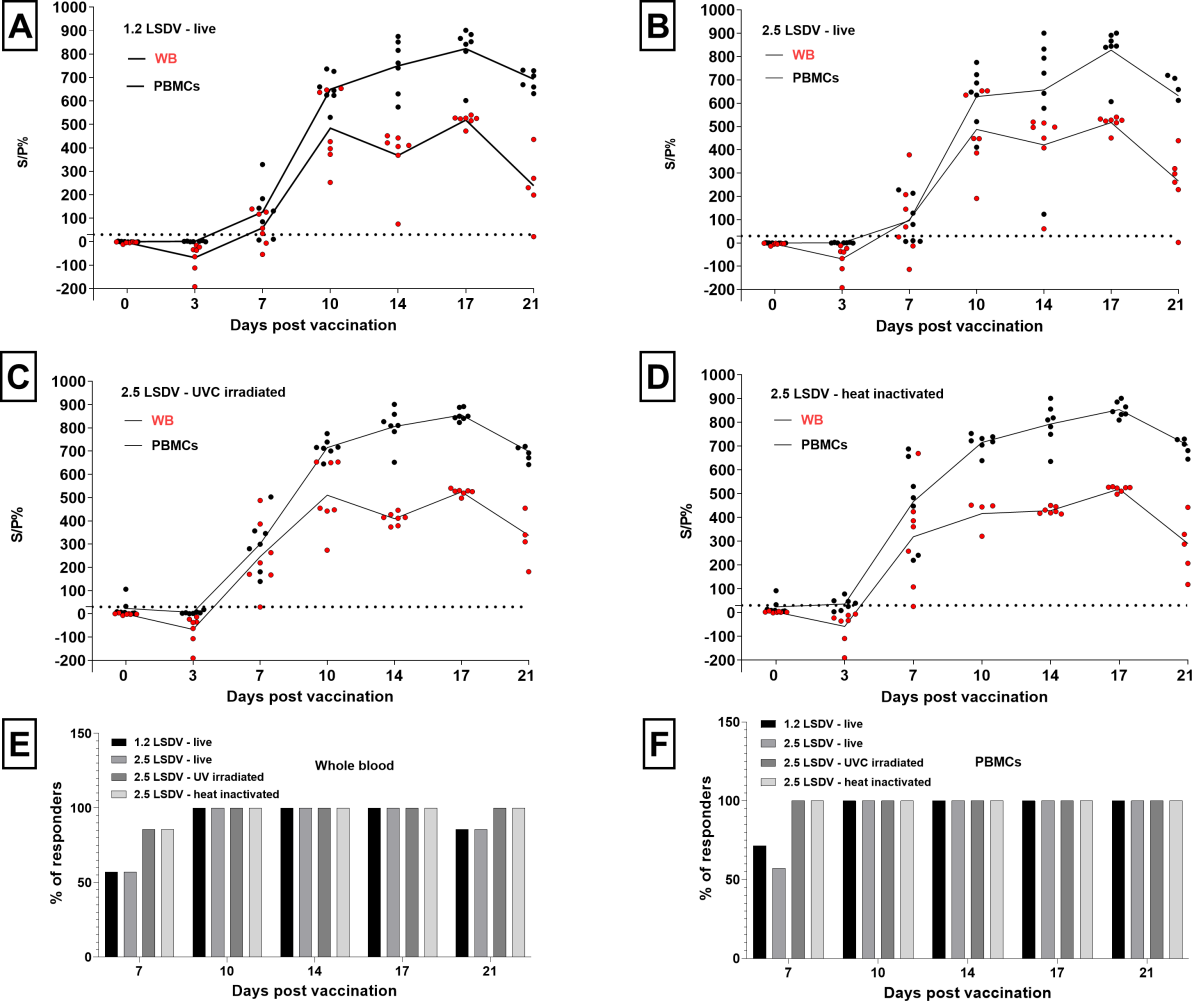
Interferon-gamma response to live attenuated Neethling-based vaccine. *In vitro* restimulated IFNg response is obtained in two matrices: whole blood and peripheral blood mononuclear cells. IFNg release is restimulated with four different stimuli: live classical clade 1.2 LSDV (A), live recombinant clade 2.5 LSDV (B), 2.5 LSDV UVC-irradiated (C) and 2.5 LSDV heat-inactivated (D). Graphs show individual values, mean is denoted in a solid line. IFNg is restimulated in interferon gamma release assay and measured with IFNg ELISA. Values are expressed as a sample to positive control ratio (S/P%). The percentage of positive animals with detectable IFNg levels in WB and PBMCs is depicted in plots (E) and (F), respectively.

IGRA allowed an early detection of a CMI response to a primo s/c Neethling-based LAV vaccination. Depending on the stimulus, between 51,74 and 100% of the animals were already found IGRA-positive at 7 dpv, as demonstrated in Fig. 2E and F, and all were positive at 10 dpv.

The IFN-γ release pattern (Fig. 2A through D) in WB and PBMCs indirectly reflects an early expansion of the antigen-specific cells detected at 7 dpv as low-to-medium IFN-γ secretion to *in vitro* restimulation. This expansion phase reached a plateau with stable IFN-γ levels detected between 10 and 17 dpv. Stable IFN-γ levels over more than 2 weeks post-vaccination are followed by signs of contraction from 21 dpv.

The start of a contraction is supported with significantly lower IFN-γ levels measured on 21 dpv compared to 17 dpv in WB (Mann–Whitney test; *P* = 0.0006 for all four stimuli) and PBMCS (Mann–Whitney test; *P* = 0.0262 for live 1.2 LSDV, *P* = 0.0175 for live 2.5 LSDV, *P* = 0.0006 for 2.5 LSDV UVC-irradiated and heat-inactivated stimuli).

#### Impact of the matrix and stimulus

Since a variation in the number of IGRA-positive animals was found at 7 dpv, we first looked at this time point in more detail.

**Fig 3:**
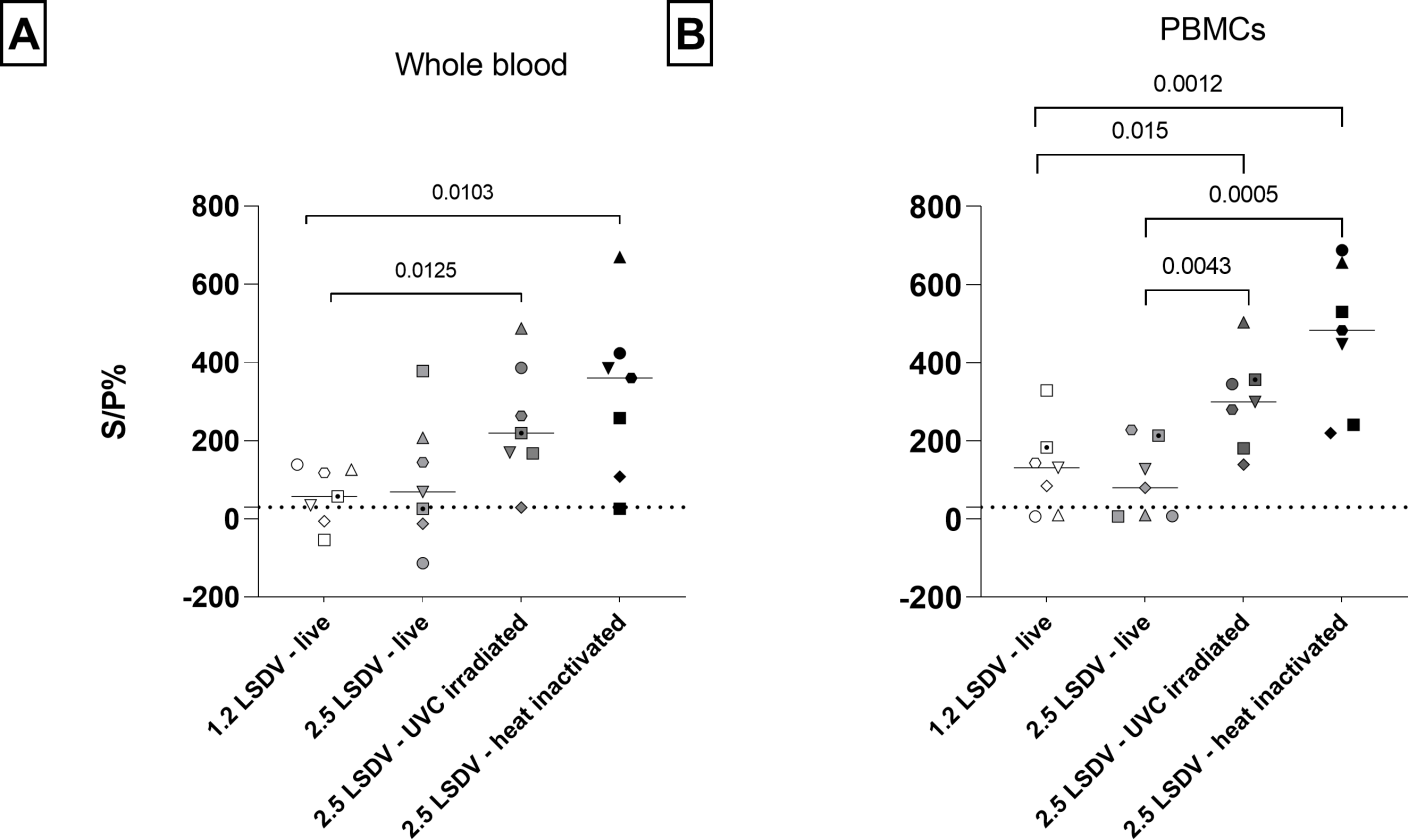
Restimulation of whole blood (A) and peripheral blood mononuclear cells (B) at 7dpv in interferon gamma (IFNg) release assay. Restimulation was performed with a live, UVC-irradiated and heat-inactivated lumpy skin disease virus from clades 1.2 and 2.5. IFNg levels are measured with IFNg ELISA and values are expressed as sample to positive control ratio (S/P %). Individual animals are depicted with different symbols. UVC-irradiated and heat-inactivated LSDV improved detection of vaccinated animals early (7dpv) post-vaccination.

At 7 dpv in WB, the IFN-γ response to the 2.5 LSDV UVC-irradiated and heat-inactivated stimuli was significantly higher than to live 1.2 LSDV (*t*-test, *P* = 0.0125 and *P* = 0.0103, respectively) (Fig. 3A). The same was found in PBMCs with a significantly higher IFN-γ response to 2.5 LSDV UVC-irradiated and heat-inactivated stimuli than to live 1.2 LSDV (*t*-test, *P* = 0,015 and *P* = 0.0012, respectively), but also to live 2.5 LSDV (*t*-test, *P* = 0.043 and *P* = 0.0005, respectively) (Fig. 3B).

These higher IFN-γ levels (and more IGRA-positive animals, see below) in both matrices in response to UVC-irradiated and heat-inactivated LSDV suggest that these inactivated stimuli could be superior compared to live LSDVs in LSD IGRA.

Subsequently, we looked at whether a similar observation could be made for the IFN-γ levels reached during the plateau phase (10–17 dpv).

Plateau IFN-γ levels in response to both live LSDVs were not significantly different in WB (Mann–Whitney test; *P* = 0.421) nor in PBMCs (Mann–Whitney test, *P* = 0.803). The same was found for UVC-irradiated and heat-inactivated LSDV in WB (Mann–Whitney test, *P* = 0.620) and in PBMCs (Mann–Whitney test, *P* > 0.512). Likewise, during the plateau, there were no significant differences between responses to live and UVC-irradiated and heat-inactivated stimuli in WB (Mann–Whitney test, *P* > 0.1) nor in PBMCs (Mann–Whitney test, *P* > 0.09).

These results indicate that there is i) no difference in IFN-γ response dependent on the stimulus when the plateau level is reached and that ii) immune cells primed by an LA Neethling-based vaccination equally react to restimulation with heterologous live, UVC-irradiated, and heat-inactivated LSDV.

A difference between IFN-γ secretion levels was, however, found between both matrices. Released amounts of IFN-γ were found to be significantly higher in PBMCs than in WB for all four stimuli (Mann–Whitney tests, *P* < 0.0001), indicating that PBMCs support increased IFN-γ secretion.

Next, we evaluated the uniformity of the response between the different animals. For each matrix and stimulus, the IFN-γ response seemed highly uniform among all animals at each time point, with the least variability between animals after stimulation with UVC-irradiated and heat-inactivated LSDV. This was explored in more detail based on the mean coefficient of variation (CV%) of each combination of matrix and stimulus.

There were no significant differences in CV% between the four stimuli in WB (Kruskal–Wallis test, *P* = 0,277) nor in PBMCs (Kruskal–Wallis test, *P* = 0.1).

Also, the average CV% during the plateau phase (from 10 to 17 dpv) between PBMCs and WB was not significantly different for any of the stimuli (multiple Mann–Whitney test, *P* > 0.7)

Mean CV% of PBMCs restimulated with the UVC-irradiated (CV = 10.78%) and heat-inactivated LSDV (CV: 10.92%) were highly similar during the plateau phase. This shows the LSDV’s ability to stimulate robust IFN-γ release *in vitro,* which is not affected by different inactivation processes and that both can be used to produce the LSD IGRA stimuli.

#### LSD IGRA sensitivity and specificity for detection of the cellular immune response post-vaccination

First, IGRA-positive animals were detected at 7 dpv. A higher number of positive animals (6/7 and 7/7 in WB and PBMCs, respectively) were detected in response to stimulation with UVC-irradiated and heat-inactivated LSDV compared to stimulation with live LSDVs (5/7 and 4/7 in WB and PBMCs, respectively) (Fig. 2E and F). Although this result indicates that the inactivated stimuli might allow a more sensitive IGRA detection of vaccinated animals early after vaccination, these ratios are not significantly different (Fisher’s exact tests, *P* > 0.05). Between 10 and 17 dpv, all animals were found positive for all stimuli, leading to maximum sensitivity.

The overall sensitivity of the assay between 7 and 21 dpv was 97.14% in WB and 100.00% in PBMCs (Fig. 1) with inactivated stimuli, and 90.00% in WB and 92.86% in PBMCs with live stimuli.

To get a preliminary idea of the specificity of the IGRA test, we looked at data from the animals (VAC; *n* = 7) before vaccination with Neethling-based LAV at 0 dpv. The specificity was calculated for each matrix and for live and UVC-irradiated and heat-inactivated LSDV (*n* = 14 samples in total per stimulus).

LSD IGRA achieved high specificity in WB regardless of the employed stimulus (Table 1). The specificity in PBMCs upon live antigen restimulation is likewise high, but UVC-irradiated and heat-inactivated LSDV induced a false-positive reaction in two animals, giving a specificity of 71.43%.

**TABLE 1:** LSD IGRA specificity

Group	Matrix	Number of samples	Sp% Live LSDV Ag	Sp% HI inactivated /UV-irradiated LSDV Ag
VAC	WB	*N* = 14	100.00%	100.00%
	PBMCs	*N* = 14	100.00%	71.43%

### Infection elicited cell-mediated immune responses

#### General observations

**Fig 4:**
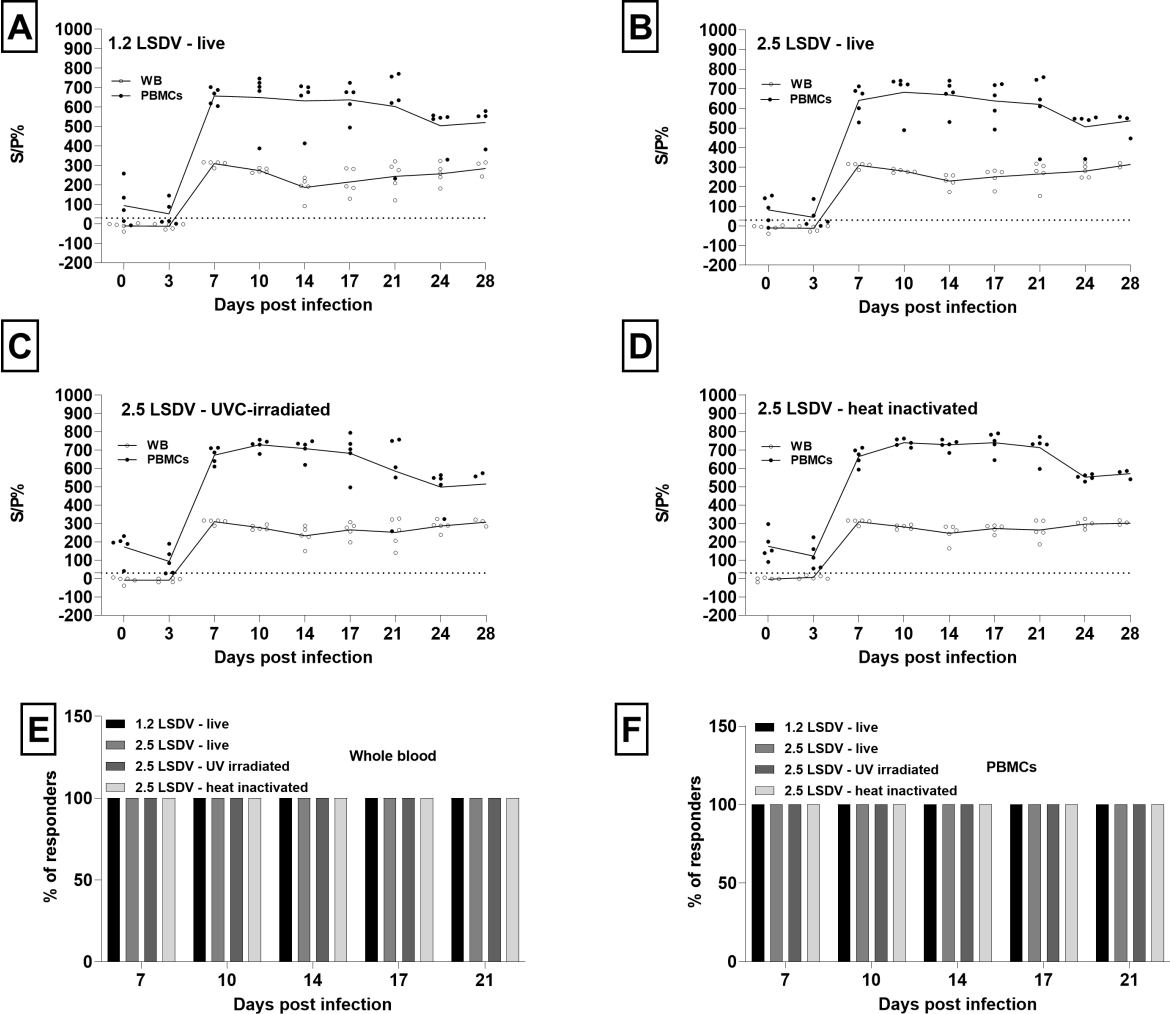
Interferon-gamma response to infection with a clade 2.5 recombinant LSDV. *In vitro* restimulated IFNg response is obtained in two matrices: whole blood and peripheral blood mononuclear cells. IFNg release is restimulated with four different stimuli: live classical clade 1.2 LSDV (A), live recombinant clade 2.5 LSDV (B), 2.5 LSDV UVC-irradiated (C), and 2.5 LSDV heat-inactivated (D). Graphs show individual values, mean is denoted in a solid line. IFNg is restimulated in interferon-gamma release assay and measured with IFNg ELISA. Values are expressed as a sample to positive control ratio (S/P%). The percentage of positive animals with detectable IFNg levels in WB and PBMCs is depicted in plots (E) and (F), respectively.

LSD IGRA also allowed early detection of a CMI response to a combined i/v and i/d inoculation of a clade 2.5 recombinant LSDV strain. In samples collected during the acute phase of infection, an *in vitro* restimulated IFN-γ response was already detected at its plateau at 7 dpi in all animals (Fig. 4A through D). Plateau IFN-γ levels are maintained constant from 7 to 28 dpi in WB, reflecting a sustained immune reaction during infection. In PBMCs, however, IFN-γ levels decreased between 17 dpi and 24 dpi, but this decrease was significant only for heat-inactivated LSDV (Mann–Whitney test, *P* = 0.0079). These results highlight that the detection of a positive IFN-γ response in IGRA might be a potential diagnostic marker of LSDV infection.

#### Impact of matrix and stimulus

Next, we looked in more detail for the differences in response to stimuli.

Plateau IFN-γ levels (7 until 28 dpi) in response to both live stimuli did not differ significantly in WB (Mann–Whitney test; *P* = 0.165) nor in PBMCs (Mann–Whitney test, *P* = 0.806). The same was found for UVC-irradiated and heat-inactivated stimuli in WB (Mann–Whitney test, *P* = 0.918) and in PBMCs (Mann–Whitney test, *P* = 0.152). Likewise, responses do not differ significantly between live stimuli and UVC-irradiated in WB (Mann–Whitney test, *P* > 0.07) and PBMCs (Mann-Whitney test, *P* > 0.192). However, plateau IFNg in response to heat-inactivated LSDV was significantly higher than to live 1.2 LSDV (Mann–Whitney test, *P* = 0.042) in WB. In PBMCs, the response to heat-inactivated LSDV was significantly higher than to live 1.2 LSDV (Mann–Whitney test, *P* = 0.0061) and live 2.5 LSDV (Mann–Whitney test, *P* = 0.011).

These results indicate immune cells primed by a clade 2.5 recombinant strain without significant differences not only cross-react to stimulation with live homologous 2.5 recombinant and heterologous live 1.2 classical strain but also to UVC-irradiated stimuli. However, heat-inactivated homologous 2.5 recombinant LSDV is a superior stimulus to live LSDV as stimuli.

When comparing IFN-γ responses in both matrices, IFN-γ levels were significantly higher in PBMCs than in WB for all stimuli tested (Mann–Whitney *P* < 0.0001 for all four stimuli), indicating that PBMCs support increased IFN-γ secretion.

Similarly to the post-vaccination response, we evaluated the uniformity of the response among animals. Overall, the responses appeared homogenous among animals at all time points in the post-infection period. We assessed this in more detail by studying CV differences in response to the four stimuli.

There were no significant differences in CVs in response to different stimuli in WB (Kruskal–Wallis test, *P* = 0,439). A significant difference was, however, found in PBMCs between the four stimuli (Kruskal–Wallis test, *P* = 0,01). *Post-hoc* tests show that the CV% of heat-inactivated LSDV was found to be significantly lower than the CV% of live 1.2 LSDV.

The average CV for all four stimuli during the plateau phase after infection (7–28 dpi) does not differ significantly between PBMCs and WB (multiple Mann–Whitney test, *P* > 0.1), indicating a similar uniformity of response in both matrices. The lowest average CV is achieved upon restimulation of PBMCs with heat-inactivated LSDV.

#### LSD IGRA sensitivity and specificity for detection of infected animals

LSD IGRA demonstrated excellent sensitivity (Fig. 4E and F) as all animals were detected positive starting from 7 dpi until 28 dpi for all stimuli in both matrices.

In WB samples collected at 0 dpi from five animals in the infected group, a high specificity was detected in response to all four stimuli, with no aspecific reactions in these samples (Fig. 4A through D).

Restimulated PBMCs collected before infection at 0 dpi reacted with low IFN-γ levels, independent of the stimulus used (Fig. 4A through D).

### Antibody response upon LSDV vaccination and infection

**Fig 5:**
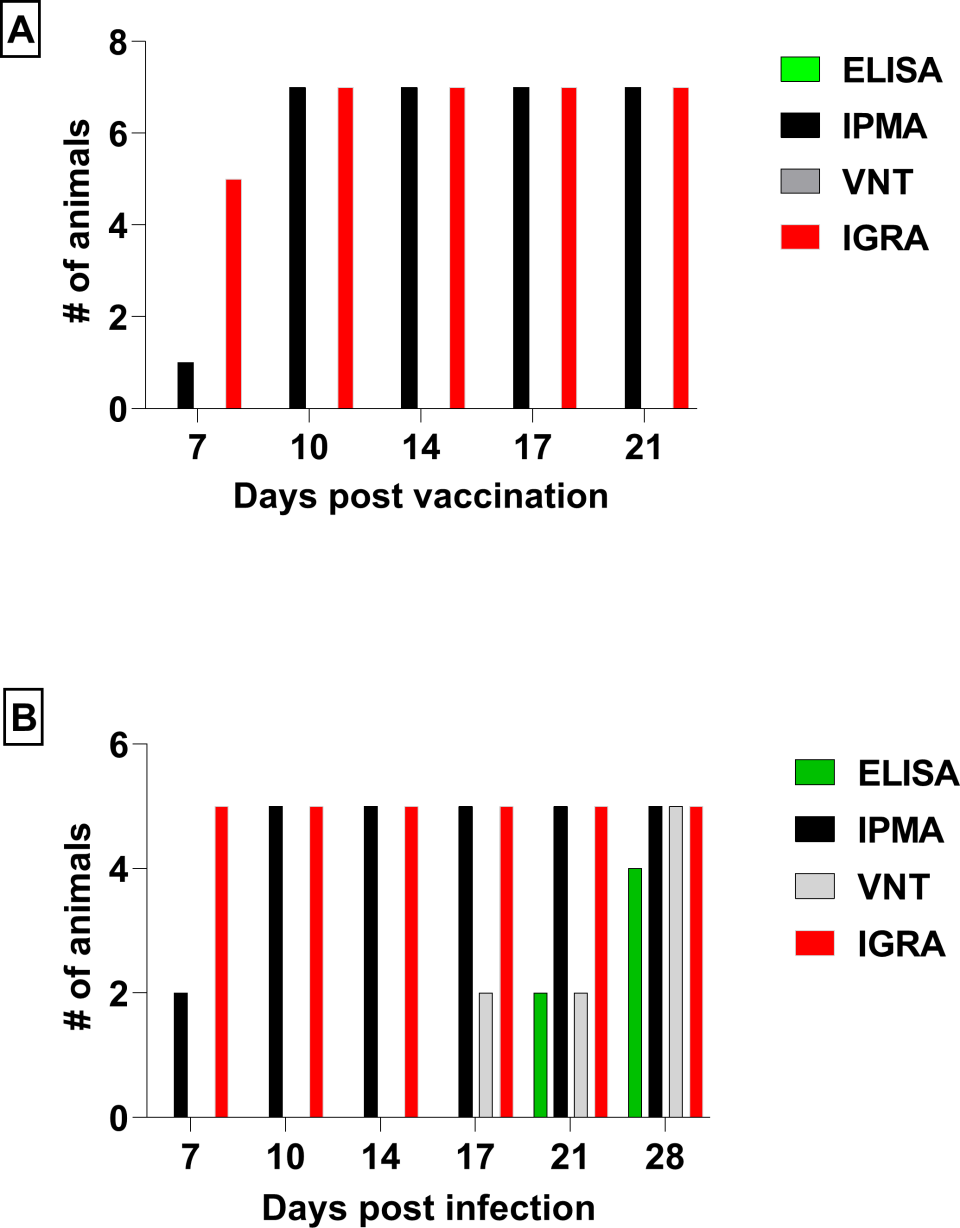
Number of animals with detected seroconversion and cell mediated immune response upon vaccination with Neethling-based live attenuated vaccine (A) and upon infection with 2.5 recombinant LSDV (B). Seroconversion was measured with three different serological tests: enzyme linked immunosorbent assay (ELISA), immunoperoxidase monolayer assay (IPMA) and virus neutralization test (VNT). Cell mediated immune response is measured with in vitro restimulated interferon gamma (IFNg) in interferon gamma release assay (IGRA) and measured with IFNg ELISA.

All animals in the vaccinated group had already seroconverted by 10 dpv, as demonstrated with IPMA (Fig. 5A). Antibodies were, however, not detected when using a commercial ELISA at any of the time points (7, 10, 17, and 21 dpv). Similarly, neutralizing antibodies were also not detected at any of these time points by virus neutralization tests (Fig. 5A)

On the contrary, 2/5 (40%) and 4/5 (80%) of infected animals were found seropositive at 21 and 28 dpi, respectively, in ELISA (Fig. 5B). Again, seroconversion was found in all animals in IPMA from 10 dpi onward (Fig. 5B). Neutralizing antibodies were found in two infected animals at 17 dpi and in all infected animals (*n* = 5) by 28 dpi.

## DISCUSSION

Currently, past or active LSD infection and the success of vaccination are investigated mainly by focusing on the humoral immune response, without looking at the CMI as an integral and crucial part of the immune reaction to LSDV. The known downsides associated with the most used serological tests (suboptimal sensitivity, long duration, and need for BSL3 labs) and the delayed or absent seroconversion against LSDV in some animals make focusing only on serological tests sometimes difficult, impairing early detection or post-vaccination monitoring.

Therefore, we aimed to learn about the CMI response upon LSDV vaccination and infection and to evaluate its full potential for use in diagnostic settings. To achieve those goals, we explored the development of the cellular and humoral immune response over 21 days upon vaccination with a Neethling-based LAV and 28 days post-LSDV infection employing IGRA, ELISA, IPMA, and VNT.

Along with the investigation of the development of the immune response, we optimized the LSD IGRA setup and evaluated i) the suitability of WB and PBMCs as the matrix for LSD IGRA, ii) the uniformity of response to vaccination and infection in each matrix, iii) the most appropriate type of stimulus for LSD IGRA, and iv) the sensitivity and specificity of LSD IGRA in detecting a CMI response to vaccination and infection over time.

Our study for the first time demonstrates the IFN-γ secretion pattern (expansion, plateau, and contraction) to a prime dose of Neethiling-based LAV, commonly used as a preventive strategy to control LSD. These new insights into LSD-related immune responses add value to previously published work on immune responses to LSDV in post-vaccination and post-infection settings (10–12).

We detected a primary post-vaccination IFN-γ response at 7 dpv, similarly to what has been previously reported (12). We also found that this primary CMI response precedes antibodies, even when the latter are determined with the sensitive IPMA test. This is in line with the initial response found after primary vaccination with vaccinia—a brisk increase in T cell response followed by the development of neutralizing antibodies (nAbs) (26). However, we did not detect nAbs upon vaccination most probably because animals were euthanized already at 21 dpv. As we demonstrated here and in line with the previous report (12), around this time, nAbs are on the rise and detected in most of the infected animals.

The magnitude and pattern of the induced IFN-γ response could potentially serve as a good first predictor of vaccine-induced protection, although LSD vaccine efficacy needs to be evaluated via virulent challenge experiments.

This primary post-vaccination IFN-γ response is sustained, with the plateau phase until 17 dpv and the beginning of the contraction phase at 21 dpv. It is, therefore, expected that the response to LAV of this quality and magnitude upon vaccination will generate a sufficient pool of LSDV-specific memory cells. This is supported by the IFN-γ memory response previously reported for LAV and inactivated LSDV vaccines. Haegeman *et al.* were able to detect a cellular memory response 12 and 18 months post-vaccination at a 100% and 80% rate, respectively (11).

In addition to revealing the characteristics of the primary post-vaccination CMI response, we also explored the CMI response post-infection. The CMI response to experimental LSDV infection was detected in all animals already at plateau levels at 7 dpi. Similarly to post-vaccination CMI, it precedes the development of antibodies in all animals of the INF group. The plateau phase post-infection is long-lasting and remains constant at least until 28 dpi. These findings confirmed previous reports on IFN-γ detection upon virulent challenge (12) and upon vector transmission from subclinical animals (8). However, previously published data contained more variation between the responses of individual animals. Based on the findings we present here, it seems that the high and constant IFN-γ response to LSD infection suggests that sustained cellular immune response contributes to the clearance of infection. The importance of CMI responses in recovery from poxvirus infections has been well-established in humans (26). This observation is further supported by the relative ineffectiveness of vaccinia antibody treatments in preventing and treating progressive vaccinia in T-cell–deficient patients (26).

Since the results above show that the CMI response upon infection and vaccination was detected early in all animals at high and constant levels using the IGRA, and it occurred even before seroconversion, it seems suitable to consider whether the *in vitro* restimulated IFN-γ response could be used as a diagnostic marker. We, therefore, evaluated different setups of the LSD IGRA, including different matrices and different stimuli for IFN-γ secretion. Both matrices provided conditions for a continuous detection of IFN-γ over time. Average post-vaccination and post-infection IFN-γ levels during the plateau were significantly higher detected from PBMCs than from WB. Hartmann *et al*. 2016 found that high PBMC concentrations (5 × 10^6^ and 10 × 10^6^) provide optimal IFN-γ response, while the response was variable between animals in WB, which suffer from physiological variation of the immune fraction (27). We observed that physiological oscillation in the immune fraction had little influence on the magnitude of response *in vitro*, in both matrices, at least when the number of cells was within the reference range.

PBMC deprivation from other blood components could affect their functionality and cause a suboptimal immune response (27). The impact on the functionality was probably neutralized by performing PBMC cultivation in autologous plasma without any treatment. This physiological medium helps better resemble *in vivo* conditions, supply IFN-γ-producing cells with the necessary costimulatory factors, and thus create the optimal conditions for secretion of IFN-γ in IGRA. It was shown that unprocessed plasma factors influence the biogenesis of phospholipids, essential membrane components, required for normal functioning of APCs, since they are involved in endocytosis, antigen processing, and presentation (28). Furthermore, plasma reuse decreased the overall costs of the assay.

Also, the variability between responders in our study was strikingly lower than in previous reports on IFN-γ (11, 12, 23). This difference could be due to differences in the immunogenicity of LAV vaccine preparations, as demonstrated before (10), but could also be influenced by the quality of blood samples and stimuli.

Next, we aimed to find the optimal stimulus to include in the IGRA test. The response detected after stimulation with two live LSDV strains, one classical (clade 1.2) and one recombinant (clade 2.5) strain, was not significantly different, probably due to the high genetic similarity between strains belonging to different clades. This suggests that the CMI response raised by LSDV infection or by vaccination with a Neethling (clade 1.1) strain results in a broad response that could confer protection against (re)infection with strains of other clades.

The promising results obtained with UVC-irradiated LSDV (12) and effective priming of CD8 cells by heat-inactivated antigens in vaccinia research (29) encouraged us to explore stimulation with UVC-irradiated and heat-inactivated LSDV in the LSD IGRA. The response to both was not significantly different, although the mean response to heat-inactivated LSDV was higher than to UVC-irradiated LSDV at 7 dpv. Heat-inactivated LSDV provided significantly higher IFN-γ levels in WB and PBMCs than both live LSDV stimuli and increased the sensitivity of the assay early post-vaccination. Therefore, heat-inactivated LSDV can be selected as the preferred stimulus for LSD IGRA. Furthermore, the use of inactivated antigens in the LSDV IGRA makes it possible to overcome the necessity to work in BSL3 conditions and implement the LSD IGRA in BSL2.

The improved performance of the heat-inactivated or UVC-irradiated antigens compared to live virus stimuli could be related to their easier presentation by APCs. Live LSDV has many antigens competing for presentation by APCs, while heat inactivation seems to decrease the number of potential viral antigens, and especially nonabundant viral proteins, by two-thirds from approximately 200 to approximately 80 in vaccinia (29), thereby increasing the chances for abundant structural proteins to be presented.

The IGRA seemed to have excellent sensitivity after both infection and vaccination. Furthermore, the IGRA performed on WB also showed excellent specificity on the limited number of samples that were tested and could be a good choice for post-infection monitoring. Some false-positive results were, however, found when using PBMCs. These problems with specificity on samples from 0 dpi are probably related to contamination during the testing. LSD IGRA is a multi-step assay, and thus errors can be introduced at any point during testing. Furthermore, IGRAs are functional assays susceptible to modulation by different factors. The sources of variation can be roughly divided into preanalytical, analytical, manufacturing, and immunological issues (30). False-positive results could be the result of contamination of antigen wells with polyclonal mitogen (30). Besides this, possible microbiological immunomodulation *ex vivo* could also have a role. It is well known that microbes contain conserved parts acting as strong modulators of innate and adaptive immunity. They can be introduced into the sample during venipuncture or by the operator during the testing. It was shown that small amounts of *Staphylococcus aureus,* which can be found on the skin, spiked into the TB antigen tube alone or into both the control and antigen tubes are sufficient to cause false-positive results in 6.9% of 58 uninfected individuals tested (31). The suboptimal specificity results obtained with PBMCs emphasize that additional validation of the test is required, including testing of a large number of field samples collected from cattle in different epidemiological situations.

In conclusion, analysis of samples collected from experimentally LSDV-vaccinated or infected cattle showed that IGRA is a sensitive test allowing early detection of exposure to LSDV antigens within 7 days after infection with a recombinant LSDV strain and 10 days after vaccination with a Neethling-based LAV.

Both the IGRA performed on WB and PBMCs provided good results, although WB might be most suitable for diagnostic purposes, considering the simplicity of working with this matrix and some observed issues with specificity in PBMCs after infection, which need further study. Also, different stimuli provided good and comparable results, but heat-inactivated antigens might be favored considering the ease of production and standardization and the potential to perform the test under BSL2 conditions.

These overall positive results support a further evaluation of the LSD IGRA specificity and sensitivity using field samples collected from animals under different epidemiological conditions. This will enable us to assess the robustness of the LSD IGRA in detecting both acute and memory responses.

Furthermore, in diagnostic applications, it is essential to determine the optimal time window between blood collection and testing to prevent loss of immune cell functionality and secure reliable results of the IGRA. The results we presented here already provided some insights into the CMI response elicited by LSDV vaccination and infection. The post-vaccination CMI response is quick and high, with a contraction phase that starts after 3 weeks. The post-infection CMI response is very quick, high, and prolonged. In each setting, the CMI response preceded antibody detection, pointing to IFN-γ secreted upon *in vitro* restimulation as a promising diagnostic marker. Finally, sensitized immune cells by Neethling-based vaccination were reactive against stimulation with both the classical and recombinant field strains, providing an indication that this LAV could confer broad protection, although the actual role in protection against (heterologous) challenge needs to be studied *in vivo*.
